# Clinically significant bleeding in incurable cancer patients: effectiveness of hemostatic radiotherapy

**DOI:** 10.1186/1748-717X-7-132

**Published:** 2012-08-03

**Authors:** Nikola Cihoric, Susanne Crowe, Steffen Eychmüller, Daniel M Aebersold, Pirus Ghadjar

**Affiliations:** 1 Department of Radiation Oncology, Bern University Hospital, and University of Bern, , , Bern, Switzerland; 2 SAKK Coordinating Center, Bern, Switzerland; 3 Center of Palliative Care, Bern University Hospital, and University of Bern, Bern, Switzerland

**Keywords:** Cancer, Bleeding, Hemostatic, Palliative, Radiotherapy

## Abstract

**Background:**

This study was performed to evaluate the outcome after hemostatic radiotherapy (RT) of significant bleeding in incurable cancer patients.

**Methods:**

Patients treated by hemostatic RT between November 2006 and February 2010 were retrospectively analyzed. Bleeding was assessed according to the World Health Organization (WHO) scale (grade 0 = no bleeding, 1 = petechial bleeding, 2 = clinically significant bleeding, 3 = bleeding requiring transfusion, 4 = bleeding associated with fatality). The primary endpoint was bleeding at the end of RT. Key secondary endpoints included overall survival (OS) and acute toxicity. The bleeding score before and after RT were compared using the Wilcoxon signed rank test. Time to event endpoints were estimated using the Kaplan Meier method.

**Results:**

Overall 62 patients were analyzed including 1 patient whose benign cause of bleeding was pseudomyxoma peritonei. Median age was 66 (range, 37–93) years. Before RT, bleeding was graded as 2 and 3 in 24 (39%) and 38 (61%) patients, respectively. A median dose of 20 (range, 5–45) Gy of hemostatic RT was applied to the bleeding site. At the end of RT, there was a statistically significant difference in bleeding (*p* < 0.001); it was graded as 0 ( *n* = 39), 1 ( *n* = 12), 2 ( *n* = 6), 3 ( *n* = 4) and 4 (n = 1). With a median follow-up of 19.3 (range, 0.3-19.3) months, the 6-month OS rate was 43%. Forty patients died (65%); 5 due to bleeding. No grade 3 or above acute toxicity was observed.

**Conclusions:**

Hemostatic RT seems to be a safe and effective treatment for clinically and statistically significantly reducing bleeding in incurable cancer patients.

## Background

Bleeding in cancer patients can occur in a variety of ways, from chronic occult bleeding to clinically significant macroscopic bleeding or profound bleeding from large blood vessels which may cause sudden death. It can be the first symptom of a disease or develop later along with disease progression. It has been estimated that bleeding occurs in approximately 6-10% of patients with advanced cancer; for at least some of these patients, bleeding will be the direct cause of death [1].

Clinically significant bleeding commonly leads to hospitalization. It is distressing to patients and their families; thus bleeding is likely to impact negatively upon patient’s quality of life (QoL). The hemostatic effectiveness of radiotherapy (RT) is usually visible after only a few fractions of RT and generally explained by increased adhesion of platelets to the vascular endothelium [2]. The long term effect could be explained by causing vessel fibrosis combined with tumor remission [3]. Although RT has been used for decades as a non-invasive treatment for cancer related bleeding, there is little published literature focusing on hemostatic RT. The reduction of hemoptysis achieved by palliative RT in non-small cell lung cancer (NSCLC) has been demonstrated within prospective randomized trials [4,5]. Moreover, several relatively small retrospective studies have described the effectiveness of hemostatic RT for vaginal bleeding caused by cervical or endometrial cancer [6,7], bleeding from locally advanced bladder cancer [8], prostate cancer [9], rectal cancer [10] and gastric cancer [11].

This study was performed to evaluate the outcome after hemostatic RT in patients who suffered clinically significant bleeding in different sites, primary tumors or involved metastases.

## Methods

### Patient selection

In this retrospective study all patients who were treated by hemostatic RT, in the Department of Radiation Oncology, Bern University Hospital, Switzerland between November 2006 and February 2010, were selected. All of these patients had advanced incurable cancer; 1 patient’s benign cause of bleeding was pseudomyxoma peritonei. Prior surgery, chemotherapy or RT was allowed as well as prior hemostatic measures. Pretreatment investigations included complete medical history, physical examination and radiological or endoscopic examination to confirm or diagnose the bleeding site, if needed. This study was approved by the local ethics committee.

### Treatment

Patients were either treated by external beam RT (EBRT), high-dose rate brachytherapy (HDR-B) monotherapy or a combination of HDR-B and EBRT. EBRT was applied using one of the following methods: a three-dimensional conformal RT (3D-CRT), a conventional two-dimensional RT or a volumetric modulated arc technique. For 3D-CRT a dedicated computed tomography (CT) scan was used for treatment planning. All treatment plans were calculated by a dedicated treatment planning system. EBRT was applied with photons from a linear accelerator. RT could also be accompanied by concomitant cisplatin based chemotherapy in certain cases.

Due to the different fractionation schedules used the dose prescriptions were translated into 3-Gy equivalent doses (EQD_3_), assuming α/β = 10, according to the linear quadratic model, with no correction for overall treatment time, as a total dose of 30 Gy using daily fractions of 3 Gy is a widely accepted and commonly used treatment schedule for palliative RT.

### Assessment and evaluations

Patients were initially seen daily by a radiation oncologist during RT, then twice weekly when the bleeding completely stopped. Due to the limited life expectancy of treated patients and their generally poor performance status (PS), patients were commonly followed-up close to their domicile either by their treating medical oncologist or family doctor. Therefore, in most cases, the patients’ condition was continuously reported. These reports together with documentation in case of further treatment in Bern University Hospital and additional information provided by the treating medical oncologist or family doctor were used to assess bleeding status at the end of follow-up and overall survival (OS).

The bleeding score was retrospectively assessed by one single observer (NC) using all available clinical information.

Bleeding was assessed before RT, the end of RT and the end of follow-up. It was graded according to the World Health Organization (WHO) scale [12] (grade 0 = no bleeding, 1 = petechial bleeding, 2 = clinically significant mild blood loss, 3 = gross blood loss requiring transfusion, 4 = debilitating blood loss associated with fatality).

OS was calculated from the last day of RT until death. Patients not experiencing an event were censored at the date of the last follow-up visit.

Toxicities were graded according to the National Cancer Institute Common Terminology Criteria for AEs (CTCAE) version 3.0. Acute toxicity was defined as toxicities occurring during or within three months after completion of RT.

### Statistical considerations

The primary endpoint was bleeding at the end of RT. Secondary endpoints included bleeding at the end of follow-up, OS as well as acute toxicity.

Bleeding before and after RT was compared using the Wilcoxon signed rank test. OS was estimated using the Kaplan Meier (KM) method. The median follow-up time was calculated using the inverse KM method, the respective range is based on patients without an event. Categorical variables were summarized using absolute and relative frequencies; continuous variables by descriptive statistics. To analyse potential associations between bleeding con-trol and clinical variables, the following variables were included: WHO bleeding score at the end of hemostatic RT and at the end of follow-up (grade 0–1 vs. grade 2–4), WHO bleeding score before hemostatic RT (grade 2 vs. grade 3), age (≤ 66 years vs. > 66 years), sex, Karnowsky PS (KPS) (≥ 50 vs. ≤ 40), histology (adenocarcinoma vs. others), presence of liver metastasis (no vs. yes), bleeding cause (primary tumor vs. metastasis), use of chemotherapy before hemostatic RT (no vs. yes), other RT prior to hemostatic RT (no vs. yes), time from first diagnosis to hemostatic RT (≥12 months vs. < 12 months), use of any other hemostatic measure (no vs. yes), dose of hemostatic RT (< 30 Gy vs. ≥ 30 Gy), and compared using the Chi-square test. Univariate and multivariate analyses for OS were performed using Cox proportional hazards models and the backward selection method (criterion for removal: *p* ≥ 0.05). To be assessed in the multivariate analysis, a variable first had to be significant (p ≤ 0.1) in the univariate analysis. The data were analyzed in SPSS (SPSS Inc., Chicago, IL, version 19.0) and SAS (Statistical Analysis Systems Institute Inc, version 9.2).

## Results

### Patient characteristics

Between November 2006 and February 2010, 62 patients were treated by hemostatic RT. Of these patients, 61 had incurable cancer disease, either due to a locally advanced primary tumor and/or presence of distant metastasis and 1 had pseudomyxoma peritonei. Median age was 66 (range, 37–93) years. Median time from the first diagnosis of the disease until start of hemostatic RT was 11 (range, 0.3-183) months. Before RT, median KPS was 40 (range, 20–80). Initial bleeding was graded as 2 and 3 in 24 (39%) and 38 (61%) patients, respectively. Six patients were lost to follow-up and hence will only be included in the evaluation of the primary endpoint. Further patient characteristics are summarized in Table 1.

**Table 1:** Patient Characteristics

**Characteristics (N=62)**		**n (%)**
Age (years)		
Median (range)	66 (37–93)	
Sex		
Female		32 (52)
Male		30 (48)
Karnowsky PS		
Median (range)	40 (20–80)	
≤ 40		32 (52)
≥ 50		30 (48)
Site*		
Bladder		10 (16)
Lung (NSCLC)		9 (15)
Endometrial		8 (13)
Prostate		6 (10)
Cervical		6 (10)
Gastric		6 (10)
Ovarial		6 (10)
Colorectal		3 (5)
Others^#^		8 (13)
**Histology**^+^		
**Adenocarcinoma**		**38 (65)**
**Squamous cell carcinoma**		**6 (10)**
**Transitional cell carcinoma**		**9 (15)**
** Others**		**6 (10)**
**Tumor-classification**^+^		
**T0**		**2 (3)**
**T3**		**2 (3)**
**T4**		**55 (94)**
Metastasis^+^		
**No**		**1 (2)**
**Yes**		**52 (88)**
**Unknown**		**6 (10)**
**Liver metastasis**^+^		
**No**		**43 (73)**
**Yes**		**16 (27)**
**Bleeding Site**^+^		
**Primary tumor**		**48 (81)**
**Metastasis**		**11 (19)**
Symptom		
Vaginal bleeding		19 (31)
Hematuria		17 (27)
Hemoptysis		10 (16)
Others		16 (26)
First diagnosis to hRT (months)		
Median (range)	11 (0.3-183)	

Abbreviations: *PS*=performance status; *NSCLC*=non-small^+^ cell lung cancer; *hRT*=hemostatic radiotherapy; ^*^Site of the primary tumor; ^#^ including esophageal (n=2), sarcoma (n=1), multiple myeloma (n=1), kidney cancer (n=1), non-hodgkin lymphoma (n=1), breast cancer (n=1) and pseudomyxoma peritonei (n=1)^+^. Three patients were not applicable having stage IV non-hodgkin lymphoma according to Ann Arbor (n=1) or stage III multiple myeloma according to Durie and Salmon (n=1) or benign pseudomyxoma peritonei (n=59).

### Treatment

Prior to treatment, 8 patients (13%) had already undergone RT to the site which was currently bleeding as part of their initial treatment, using a median dose of 61.5 (range, 10–70) Gy. Median time between primary RT and hemostatic RT in these patients was 32 (range, 2–128) months. Thirty-three patients (53%) received prior chemotherapy; 8 (13%) of which received chemotherapy within 1 month prior to hemostatic RT. Three patients (5%) took anticoagulants, of whom 2 continued this medication during and after hemostatic RT. Thirteen patients (21%) had already undergone the following hemostatic measures prior to hemostatic RT: vaginal tamponade (*n* = 4), cauterization ( *n* = 4), tumor resection ( *n* = 2), embolization ( *n* = 2) and infiltration with adrenaline ( *n* = 1). However, these measures were not successful, thus the patients were referred to RT. Hemostatic RT was then given to the bleeding site. Further information on treatment is summarized in Table 2.

**Table 2:** Treatment characteristics

**Characteristics (N=62)**		**n (%)**
Chemotherapy before hRT		
No		29 (47)
Yes		33 (53)
Time last ChT to hRT (months)		
Median (range)	3 (0.2-43)	
Chemotherapy during hRT		
No		60 (97)
Yes		2 (3)
Other RT prior hRT*		
No		54 (87)
Yes		8 (13)
Time last RT to hRT (months)		
Median (range)	32 (2–128)	
Other hemostatic measure		
No		49 (79)
Yes		13 (21)
Total hRT dose (Gy)		
Median (range)	20 (5–45)	
Region treated by hRT		
Uterovaginal		19 (31)
Bladder		17 (27)
Lung		10 (16)
Upper GI		10 (16)
Other^#^		6 (10)
Treatment time hRT (days)		
Median (range)	8 (1–25)	

Abbreviations: *RT*=radiotherapy; *hRT*=hemostatic radiotherapy; *ChT*=chemotherapy; *GI*=gastrointestinal; ^*^in the same region as hemostatic *RT*; ^#^including lower gastrointestinal (n=4), skin (n=1) and abdominal wall (n=1).

Sixty patients (97%) were treated by EBRT alone using either 3D-CRT (n = 58), conventional two-dimensional RT (n = 1) or a volumetric modulated arc technique (n = 1). The different fractionation schedules used are summarized in Table 3. In 13 patients, the single dose was adapted during RT for a variety of reasons. Median single dose by EBRT for the remaining 47 patients was 3 (range, 2–8) Gy, 1 fraction prescribed per day, 5 days per week. Of the remaining patients, 1 with esophageal cancer was treated by HDR-B monotherapy (2 x 5 Gy) and the other with cervical cancer was treated with a combination of HDR-B (2 x 7 Gy) and 3D-CRT (5 x 4 Gy).

**Table 3:** External beam radiotherapy fractionation schedules and the respective 3-Gy equivalent doses

**Fractionation schedule**	Number of patients in each schedule (N=47)* n (%)	**Total dose (Gy)**	**3-Gy ED (Gy) (α/β=10)**
10 x 2 Gy	5 (11%)	20	18.5
15 x 2 Gy	2 (4%)	30	27.7
12 x 2 Gy	1 (2%)	24	22.1
18 x 2.5 Gy	2 (4%)	45	43.3
16 x 2.5 Gy	1 (2%)	40	38.5
10 x 3 Gy	10 (21%)	30	30.0
13 x 3 Gy	2 (4%)	39	39.0
5 x 3 Gy	2 (4%)	15	15.0
9 x 3 Gy	1 (2%)	27	27.0
4 x 3 Gy	1 (2%)	12	12.0
5 x 4 Gy	12 (26%)	20	21.5
4 x 4 Gy	2 (4%)	16	17.2
6 x 4 Gy	1 (2%)	24	25.8
3 x 4 Gy	1 (2%)	12	12.9
5 x 5 Gy	1 (2%)	25	28.8
4 x 5 Gy	1 (2%)	20	23.1
1 x 5 Gy	1 (2%)	5	5.8
1 x 8 Gy	1 (2%)	8	11.1

Abbreviations: *ED*= equivalent dose; ^*^ in 13 patients the single dose was adapted during RT and 2 patients underwent high-dose rate brachytherapy.

Median total dose to the bleeding site by EBRT (excluding the patient with HDR-B monotherapy) was 20 (range, 5–45) Gy. Median RT duration was 8.5 (range, 1–25) days. RT was accompanied by concomitant cisplatin based chemotherapy in 2 patients (3%) with NSCLC.

### Bleeding control at the end of RT

**Figure 1:**
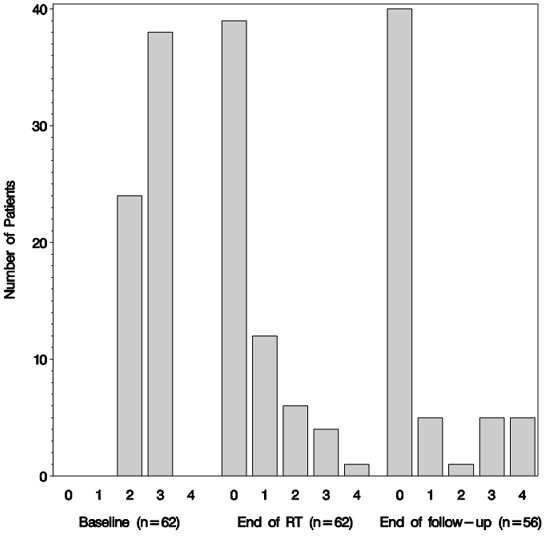
Bar chart showing the proportion of patients with WHO bleeding grade 0–4 before hemostatic radiotherapy, at the end of radiotherapy and at the end of follow-up.

At the end of RT, the following bleeding grades were observed in patients: 0 (*n* = 39), 1 ( *n* = 12), 2 ( *n* = 6), 3 ( *n* = 4) and 4 ( *n* = 1). It should be noted that 8 patients with either grade 2 or 3 at the end of RT were treated with less than 30 Gy. The incidence of bleeding was statistically significantly different from the bleeding status prior to hemostatic RT ( *p* < 0.001) (Figure 1).

**Figure 2:**
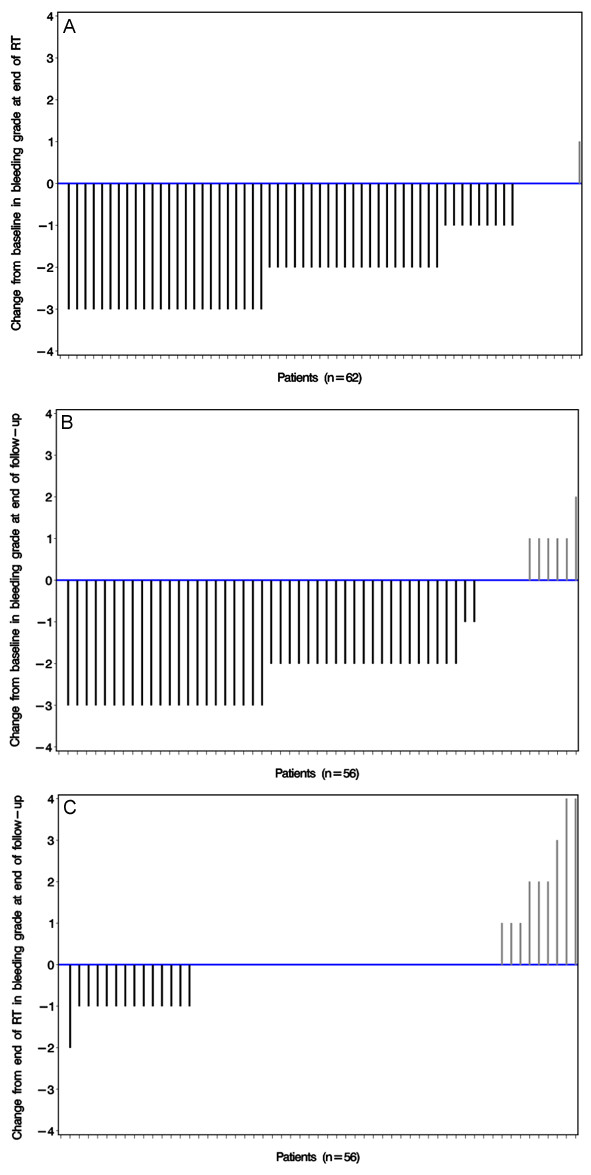
Waterfall plot showing change in bleeding grade at end of RT as compared to baseline (A), change in bleeding grade at end of follow-up as compared to baseline (B) and change in bleeding grade at end of follow-up as compared to at end of RT (C), <0 = Improvement; 0 = Stable (no change); >0 = Worsened.

Bleeding was improved in 54 patients (87%); 39 (63%) of whom had a complete response (CR) to bleeding. Of the remaining patients, bleeding was unchanged in 7 patients (11%) whilst 1 patient showed a progression in bleeding (Figure 2). This elderly gastric cancer patient died as a result of bleeding, after completion of 5 fractions reaching a total dose of 9.4 Gy (first fraction 1.4 Gy due to technical problems, followed by 4 x 2 Gy). The patient was originally referred to RT after repeated non-successful cauterizations with grade 3 bleeding.

Using the Chi-square test, sex was found to be associated with worse bleeding control at the end of hemostatic RT (*p* = 0.0253). However, age, KPS, histology, presence of liver metastasis, use of chemotherapy or other RT prior to hemostatic RT, time from first diagnosis to hemostatic RT, bleeding cause (primary tumor vs. metastasis), WHO bleeding score before hemostatic RT, use of any other hemostatic measure and dose of hemostatic RT (< 30 Gy vs. ≥ 30 Gy) were not significantly associated with bleeding control at the end of RT. When the response of bleeding at the end of RT was analyzed according to the respective bleeding site, it indicated that lesions in the lung (100% improved), uterovaginal lesions (95% improved) and upper gastrointestinal (GI) lesions (90% improved) showed a better response than lesions involving the bladder (65% improved) (Table 4).

**Table 4:** Bleeding control by treated site

**Treated Site (N)**	**Change in grade**	**Baseline to End of RT n (%)**	**Baseline to End of follow-up n (%)**	**End of RT to End of follow-up n (%)**
Uterovaginal (N=19)	Worsened	-	1 (5.3%)	2 (10.5%)
	No change	1 (5.3%)	1 (5.3%)	10 (52.6%)
	Improved	18 (94.7%)	15 (78.9%)	5 (26.3%)
	Missing	-	2 (10.5%)	2 (10.5%)
Bladder (N=17)	Worsened	-	2 (11.8%)	5 (29.4%)
	No change	6 (35.3%)	4 (23.5%)	8 (47.1%)
	Improved	11 (64.7%)	10 (58.8%)	3 (17.6%)
	Missing	-	1 (5.9%)	1 (5.9%)
Lung (N=10)	Worsened	-	-	-
	No change	-	-	6 (60.0%)
	Improved	10 (100.0%)	8 (80.0%)	2 (20.0%)
	Missing	-	2 (20.0%)	2 (20.0%)
Upper GI (N=10)	Worsened	1 (10.0%)	3 (30.0%)	2 (20.0%)
	No change	-	-	4 (40.0%)
	Improved	9 (90.0%)	6 (60.0%)	3 (30.0%)
	Missing	-	1 (10.0%)	1 (10.0%)
Other (N=6)*	Worsened	-	-	-
	No change	-	-	5 (83.3%)
	Improved	6 (100.0%)	6 (100.0%)	1 (16.7%)

Abbreviations: ^*^including lower gastrointestinal (n=4), skin (n=1) and abdominal wall (n=1).

### Bleeding control at end of follow-up

At the end of follow-up, the incidence of bleeding was as follows: grade 0 (*n* = 40), grade 1 ( *n* = 5), grade 2 ( *n* = 1), grade 3 ( *n* = 5) and grade 4 ( *n* = 5). This was statistically significantly different from the bleeding status prior to hemostatic RT ( *p* < 0.001, worse = 6, no change = 5, improved = 45) (Figure 1).

The following grades were observed at the end of follow-up for the 39 patients with no bleeding (grade 0) at the end of RT: 0 (*n* = 26), 1 ( *n* = 2), 2 ( *n* = 1), 3 ( *n* = 1) and 4 ( *n* = 2). At the end of follow-up, the 12 patients with grade 1 bleeding at the end of RT either had grade 0 ( *n* = 11) or 1 ( *n* = 1) bleeding.

From end of RT to end of follow-up, for those who could be assessed, the grade worsened in 9 patients, remained unchanged in 33 and improved in 14 (Figure 2). The change in grade in bleeding scores at end of follow-up from the end of RT were analyzed according to the respective bleeding site. Lesions involving the bladder (29% worsened) and upper GI (20% worsened) appeared to have worse scores than lung (0% worsened) or uterovaginal lesions (10.5% worsened) (Table 4).

### Overall survival

Four patients (6%) died during RT when the prescribed dose was not completely delivered for the following reasons: bleeding (grade 4) from the irradiated volume (n=1), kidney failure (n=2) and septicemia (n=1). A further 36 patients (58%) died during follow-up; 4 (6%) of which were attributed to bleeding from the treated site.Median time to death based on data from all patients was 2.4 (range, 0.03-30.9) months.

**Figure 3:**
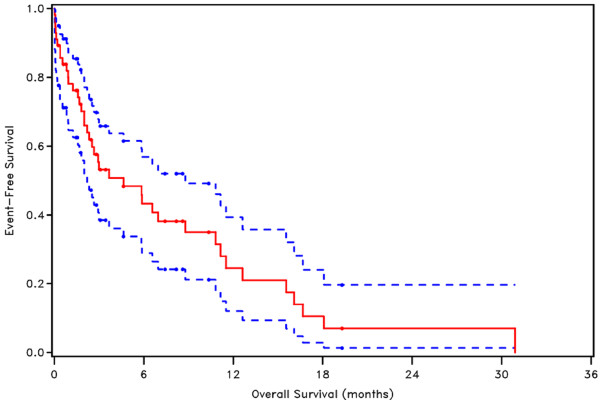
Kaplan Meier plot of overall survival with 95% confidence interval

With a median follow-up of 2.4 (range, 0.03-30.9) months, the 6-month, 1-year and 2-year OS rates were 43%, 24% and 7%, respectively (Figure 3).

In the univariate Cox proportional hazards model, sex was associated with OS (hazard ratio (HR): 1.967; 95% Confidence interval (CI): 1.033-3.748; *p* = 0.0396); male patients have a greater risk of dying. RT dose was also associated with OS (HR: 2.669; 95% CI: 1.286-5.540; *p* = 0.0084); patients who receive a low RT dose (< 30 Gy) are more likely to die. This was also the case when the same dose cut-off was used based on the EQD_3_ (HR: 2.621; 95% CI: 1.228-5.592; *p* = 0.0127). Furthermore, bleeding grade at the end of RT was associated with OS (HR: 5.882; 95% CI: 2.445-13.865; *p* < 0.0001), patients with bleeding grade ≥ 2 are shown to have a greater risk of dying. In the multivariate analysis higher bleeding grade at the end of RT and lower RT dose remained statistically significantly associated with decreased OS (Table 5).

**Table 5:** Overall survival analysis

**Dichotomized variables**	**Associated level**	**OS**	
		**Hazard ratio (95% CI)**	**p-value**
*Univariate analysis*			
Age	> 66 years	0.971 (0.514-1.834)	0.9288
Sex	Male	1.967 (1.033-3.748)	0.0396
KPS	> 50	0.539 (0.283-1.029)	0.0611
Histology	Adenocarcinoma	0.965 (0.510-1.825)	0.9118
Presence of liver metastasis	Yes	0.982 (0.463-2.082)	0.9616
Use of chemotherapy before hRT	Yes	1.085 (0.572-2.057)	0.8034
Time from first diagnosis to hRT	≥ 12 months	0.804 (0.424-1.524)	0.5037
Other RT prior to hRT	Yes	1.411 (0.656-3.034)	0.3785
Use of any other hemostatic measure	Yes	1.056 (0.409-2.724)	0.9105
Bleeding cause	Primary tumor	1.420 (0.623-3.236)	0.4036
WHO bleeding score before hRT	Grade 3	1.380 (0.728-2.617)	0.3239
Dose of hRT	< 30 Gy	2.669 (1.286-5.540)	0.0084
WHO bleeding score at end of hRT	Grade 2-4	5.882 (2.445-13.865)	<0.0001
WHO bleeding score at end of follow-up	Grade 2-4	2.006 (0.969-4.154)	0.0609
*Multivariate analysis*			
Sex	Male	1.007 (0.487-2.081)	0.9859
Dose of hRT	< 30 Gy	2.853 (1.360-5.987)	0.0056
WHO bleeding score at end of hRT	Grade 2-4	6.456 (2.645-16.202)	<0.0001

Abbreviations: *RT*=radiotherapy; *hRT*=hemostatic radiotherapy; *KPS*=Karnowsky performance score; *OS*=overall survival.

### Acute toxicity

Six patients (10%) experienced grade 1 acute toxicity; observed symptoms included diarrhea, esophagitis, abdominal pain and vaginitis. Three patients (5%) had grade 2 acute toxicity with symptoms such as esophagitis, nausea and erythema. No grade 3 or higher acute toxicities were observed.

## Discussion

We are not currently aware of any established standard therapeutic approach for the treatment of advanced cancer patients with clinically significant bleeding as a result of their cancer. At the moment, treatment decisions are primarily based on the underlying causes and overall patient PS. The therapeutic approach varies from comfort measurement to invasive surgical procedures.

Hemostatic RT is generally believed to be an effective treatment for patients with bleeding due to cancer. Although bleeding occurs in 6-10% of patients with advanced cancer [1], relatively little published literature is available which focuses on bleeding control of hemostatic RT; even less is known about the optimal RT total dose and fractionation.

In our retrospective study containing 62 patients with different primary tumors with significant bleeding (including 1 patient whose benign cause of bleeding was pseudomyxoma pertinonei), it was demonstrated that hemostatic RT reduced the incidence and grading of bleeding significantly. This effect seemed to be durable in the majority of patients. Furthermore, our data suggests an association between a total RT dose > 30 Gy and increased OS. Male patients and those with a bleeding grade ≥ 2 at the end of RT were shown to have a greater risk of dying. Although RT dose remained significantly associated with OS in the multivariate analysis, we are aware that this association may be biased due to possible confounding factors, such as increased dose given to patients with a higher PS and/or better prognosis.

Bleehen et al. reported almost 20 years ago results from a phase III trial which compared 2 different fractionation schedules (1 x 10 Gy vs. 2 x 8.5 Gy) as palliative treatment in 235 patients with incurable locally advanced NSCLC. One of the symptoms of interest was hemoptysis which was present in some form in 47% of all patients. After thoracic RT, hemoptysis improved in 72-75% of patients and completely disappeared in 54-64% of patients. Median duration of this palliative effect was 64–73 days; no significant difference was observed between the 2 arms [4]. Similarly, Langendijk et al. prospectively assessed the influence of palliative thoracic RT using 10 x 3 Gy in 65 patients with incurable NSCLC on QoL. There was a 79% response rate of hemoptysis after RT [5]. Comparably, the symptoms of all 10 patients where lung was the treated site in our retrospective study improved during RT. There was a 80% CR rate for bleeding. Furthermore, bleeding remained improved in 80% of patients until the end of follow-up.

Biswal et al. reported a bleeding control of 100% 12-48 hours after EBRT with 5–20 Gy with/or low-dose rate brachytherapy with 30 Gy for severe refractory bleeding caused by cervical cancer. However, in 85% bleeding was observed again within the following two years. GI toxicities ≥ grade 2 were observed in 3 patients [7]. Of the 19 patients who received RT to the uterovaginal region in our study, improvement of bleeding during RT occurred in 95%, 68% had a CR and bleeding remained improved until the end of follow-up for 79%. Srinivasan et al. treated in total 41 cT3-4 patients, with bladder cancer who had hematuria, with 2 x 8.5 Gy = 17 Gy (EQD_3_ = 24 Gy) as compared to 12 x 3.75 Gy = 45 Gy. Interestingly, the effect on hematuria was larger in the 17 Gy hypofractionated patients with a 59% clearing probability as compared to 16% in the other group, while toxicity did not differ between the groups [8]. Others have reported that 6 weeks after palliative RT using 20 Gy in 5 fractions, the bleeding response rate was 81% in 31 patients with castrant resistant prostate cancer, however the response rate appeared to drop to 42% and 29% after 4 months and 7 months, respectively [9]. Seventeen patients in our study received RT to the bladder region (for different primary tumors and histologies, respectively); 65% showed an improvement in their bleeding during RT, 47% had a CR and 59% remained with improved symptoms until the end of follow-up.

Hoskin et al. described the outcome after intraluminal HDR-B for 50 patients with inoperable cancer of the rectum or anal canal. Treatment was either performed in curative intent with 6 x 6 Gy = 36 Gy HDR-B monotherapy, 12 Gy HDR-B boost after 45 Gy EBRT (n = 22), or 1 x 10 Gy HDR-B as palliative treatment (n = 28). Twenty-eight patients had initial bleeding; a 57% CR rate was obtained in these patients, with a 10-month median response duration [10]. In our study only 4 patients with tumors of the lower GI tract were available, all of whom experienced CR for bleeding which remained until the end of follow-up.

Hemostatic RT was also used to treat locally advanced or recurrent gastric cancer. Tey et al.’s study contained 33 patients; 24 of whom had initial bleeding. After RT where 10 x 3 Gy was applied in the majority of cases, 54% showed a reduction in bleeding with median response duration of 140 days. No dose–response relationship was observed. One patient experienced grade 3 toxicity [11]. In our study, 10 patients received RT to the upper GI tract; 5 of whom was to the stomach. A reduction in bleeding was observed in 90%, CR in 50% and durable response in 60%. Notably in 30% of these patients bleeding worsened during the end of follow-up.

We are aware of the limitations within our retrospective non-randomized study; no firm conclusions can be made in regard to the optimal radiation dose. The possible advantages of using a higher RT dose should be balanced against potentially higher toxicity in addition to patient discomfort due to the prolonged treatment time. Hypofractionation with large single doses may be useful in case of life-threatening bleeding to induce rapid hemostypsis, but the risks of severe toxicities (Grade 3–5) have to be taken into account even in this palliative end-of-life setting [13].

We also acknowledge the heterogeneity of our patient population; several different primary tumors were involved and ultimately different treatment sites were targeted by hemostatic RT. While, the total response rate compares well with the current literature for different individual disease types, the bleeding control seemed to be lower in lesions involving the bladder as compared to the other treated sites. This finding, however, is again subject to several potential confounding factors such as use of different total doses and therefore needs further confirmation by other studies.

## Conclusions

Our data focusing on bleeding control in advanced cancer patients strongly suggests that hemostatic RT is a very effective treatment for significantly reducing bleeding of various primary tumors and treatment sites without major toxicity.

## Abbreviations

CI, Convidence interval; CR, Complete response; CT, Computed tomography; CTCAE, Common Terminology Criteria for AE; EBRT, External beam radiotherapy; EQD3, 3-Gy equivalent doses; GI, Gastrointestinal; HDR-B, High-dose rate brachytherapy; HR, Hazard ratio; KM, Kaplan Meier; KPS, Karnowsky performance status; NSCLC, Non-small cell lung cancer; OS, Overall survival; QoL, Quality of life; RT, Radiotherapy; PS, Performance status; HRT, Hemostatic radiotherapy; WHO, World Health Organization; 3D-CRT, Three-dimensional conformal radiotherapy.

## Competing interests

The authors declare that they have no competing interests.

## Authors’ contributions

Each author had participated sufficiently in the work to take public responsibility for appropriate portions of the content. NC, PG and DMA designed the study. SC and PG performed the statistical analysis. NC and PG collected the data and together with SE and DMA interpreted the data. The manuscript was written by NC and PG; all other authors helped. All authors have read and approved this manuscript.
